# Comparative Transcriptomic Study of Muscle Provides New Insights into the Growth Superiority of a Novel Grouper Hybrid

**DOI:** 10.1371/journal.pone.0168802

**Published:** 2016-12-22

**Authors:** Ying Sun, Yu Huang, Guojun Hu, Xinhui Zhang, Zhiqiang Ruan, Xiaomeng Zhao, Chuanyu Guo, Zhujing Tang, Xiaofeng Li, Xinxin You, Haoran Lin, Yong Zhang, Qiong Shi

**Affiliations:** 1 State Key Laboratory of Biocontrol, Institute of Aquatic Economic Animals and Guangdong Provincial Key Laboratory for Aquatic Economic Animals, School of Life Sciences, Sun Yat-Sen University, Guangzhou, China; 2 Shenzhen Key Lab of Marine Genomics, Guangdong Provincial Key Lab of Molecular Breeding in Marine Economic Animals, BGI, Shenzhen, China; 3 Center for Marine Research, School of Life Sciences and Oceanography, Shenzhen University, Shenzhen, China; University of California, Davis, UNITED STATES

## Abstract

Grouper (*Epinephelus spp*.) is a group of fish species with great economic importance in Asian countries. A novel hybrid grouper, generated by us and called the Hulong grouper (Hyb), has better growth performance than its parents, *E*. *fuscoguttatus* (Efu, ♀) and *E*. *lanceolatus* (Ela, ♂). We previously reported that the GH/IGF (growth hormone/insulin-like growth factor) system in the brain and liver contributed to the superior growth of the Hyb. In this study, using transcriptome sequencing (RNA-seq) and quantitative real-time PCR (qRT-PCR), we analyzed RNA expression levels of comprehensive genes in the muscle of the hybrid and its parents. Our data showed that genes involved in glycolysis and calcium signaling in addition to troponins are up-regulated in the Hyb. The results suggested that the activity of the upstream GH/IGF system in the brain and liver, along with the up-regulated glycolytic genes as well as ryanodine receptors (RyRs) and troponins related to the calcium signaling pathway in muscle, led to enhanced growth in the hybrid grouper. Muscle contraction inducing growth could be the major contributor to the growth superiority in our novel hybrid grouper, which may be a common mechanism for hybrid superiority in fishes.

## Introduction

Grouper (*Epinephelus spp*.) is a group of important economic marine fish species that are widely cultured in China and Southeast Asian countries [1, 2]. However, some threats, such as germplasm resource degradation and insufficiency of high-quality gametes in hatcheries, restrict the development of grouper aquaculture [3]. One critical way to solve these problems is hybridization technology, which has been widely applied to grouper artificial breeding over the past few decades [4–9]. A novel hybrid grouper (Hyb), generated by us and called the Hulong grouper, exhibits a faster growth rate and stronger disease resistance than its parents (*E*. *fuscoguttatus*, Efu ♀× *E*. *lanceolatus*, Ela ♂) [10–13]. Although the biological and physiological features of this hybrid species have been well-studied, the genetic mechanisms of its heterosis, especially its superior growth, have yet to be clarified.

Muscle growth is a complex process that includes cell proliferation, differentiation and material metabolism [14]. In mammals, the total skeletal muscle fiber number is determined during the embryonic period, and muscle hypertrophy occurs only after birth [15–17]. During the whole life cycle of teleosts, however, hypertrophic and hyperplastic muscle growth can take place concomitantly [18, 19]. For this reason, fish are excellent experimental models for studying fundamental growth regulatory mechanisms in vertebrates. Additionally, compared to the other vertebrates, fish muscle mass accounts for a higher proportion of body weight, which reaches 50%-70% in some economically important species [20]. Therefore, understanding the mechanisms controlling muscle growth in fish is necessary to optimize aquaculture.

Our previous research on the brain and liver of the Hyb and its parents demonstrated that the growth hormone/insulin-like growth factor (GH/IGF) axis and its downstream signaling pathways, including glycogen synthesis, contributed to the enhanced growth of the hybrid grouper [21]. Therefore, because muscle is a target of GH/IGF [22, 23], we chose to investigate the downstream glycolysis pathway and explore the genetic mechanisms underlying the superior growth in the Hyb. The other reason why we chose to examine glycolysis is that it plays a central role in the anabolism and catabolism of organisms [24] and provides intermediates for other metabolic pathways [25–27] and energy for life activities [28]. Glycolysis has been shown to be related to muscle growth. In mammals, loss of glycolytic enzymes has been observed in some skeletal muscle diseases such as inclusion body myositis [29], inflammatory myopathy [30], Duchenne muscular dystrophy [31], Becker muscular dystrophy [32] and limb weakness [33]. In teleosts, such as the model organism zebrafish (*Danio rerio*), glycolytic genes such as *Pgam*, *Pfk*, *Gapdh*, *Pgk* and *Pyk* were synchronously activated in embryonic muscles and attenuation of their expression led to reduced fiber size and unfused myoblasts [34].

Muscle growth depends heavily on high-quality muscle contractions [35, 36], which are controlled by the release of calcium into the cytosol [37]. Therefore, we further investigated, in addition to glycolysis, if other pathways, such as the calcium signaling pathway, also contributed to the enhanced growth of Hyb. Ca^2+^ release mediated by ryanodine receptors (RyRs) from the sarcoplasmic reticulum (SR) into the cytosol causes a very rapid and dramatic increase in the cytoplasmic calcium concentration, which has been widely exploited for signal transduction [38–41]. Troponin C (TnC), which contains four calcium-binding EF hands [42, 43], is one of the proteins that can directly bind to and sense Ca^2+^ [37, 44, 45]. Together with troponin I (TnI) and troponin T (TnT), TnC forms a troponin complex that binds to G-actin and tropomyosin synchronously then plays a major role in Ca^2+^ dependent regulation of muscle contraction [46, 47]. Ca^2+^ concentration variations regulated by RyRs are reported to be involved in muscle development. In zebrafish, calcium signaling via RyR channels regulated muscle fiber formation in embryos [48], and depletion of RyR1 or RyR3 caused slow muscle defects and resulted in disrupted, non-uniform slow muscle fibers [49]. In addition, complete loss of TnT activity led to sarcomere loss and myocyte disarray [50, 51] and injection of TnC morpholinos resulted in a loss of skeletal muscle function [52].

In our previous study, transcriptome sequencing (RNA-seq) of the brain and liver tissues from the Hyb and its parents demonstrated that the GH/IGF system in addition to the protein and glycogen synthesis pathway contributed to the enhanced growth in the Hyb [21], but the performance of target organs (for example, muscle) has not been reported. In this study, we sequenced the transcriptomes of muscles from the three groupers mentioned above, and the results were further validated using quantitative real-time PCR (qRT-PCR). We found that, among the annotated candidates, 8 out of the 10 enzymes catalyzing glycolysis were up-regulated in the hybrid grouper compared to its parents. In addition, the calcium signaling pathway was also highly activated and the expressions of troponins, including TnC, TnT and TnI were much higher. These findings enhanced our understanding of pathways regulating muscle development and heterosis in fish.

## Materials and Methods

### Fish and sample preparation

*E*. *fuscoguttatus* (♀), *E*. *lanceolatus* (♂) and their hybrid F1 offspring (the Hulong grouper) were cultivated under the same breeding conditions (i.e., maintained in a laboratory recirculating seawater system at 25–30°C) in the Daya Bay Seawater Fish Farm in Huizhou, Guangdong Province, China [21]. Three individuals from each species were randomly selected at the age of 18 months. Fresh muscle tissues were dissected from each fish after euthanasia by immersion in MS-222 buffered solution (3 g/L) on ice, and the tissues were immediately deposited in separate sterile tubes soaked in liquid nitrogen. The research protocol and the procedure for handling experimental animals were reviewed and approved by the Institutional Review Board on Bioethics and Biosafety of BGI.

### RNA extraction and high-throughput sequencing

Total RNA from nine muscle samples was separately isolated with Trizol reagent (Invitrogen, Carlsbad, CA, USA) and purified using the RNeasy Animal Mini Kit (Qiagen, Valencia, CA). RNA quality was then assessed using an Agilent 2100 Bioanalyzer (Agilent Technologies, Palo Alto, Calif). An equal amount of total RNA with a 28S/18S ratio ≥ 1.0 and RNA Integrity Number (RIN) ≥ 7.0 from three individuals in each group was pooled together to construct cDNA libraries (Illumina, San Diego, CA). These libraries were subsequently sequenced through an Illumina HiSeq2000 platform at BGI (Shenzhen, China). Approximately 5 Gb of raw reads were generated for each of the three samples.

### Raw reads processing, RPKM calculation and GO enrichment

Adaptor sequences and raw reads with a more than 10% N bases and quality score below 20 were filtered out using SOAPnuke [53]. The remaining clean reads were then aligned to the available genome of *E*. *coioides* (from our unpublished data) with SOAPaligner 2 [54]. The Cuffdiff package of Cufflink software (version 2.1.1) [55], with the core parameters (—FDR 0.05—geometric-norm TRUE—compatible-hits-norm TRUE) to reduce certain types of bias caused by differential amounts of RNA reads, was used to calculate the RPKM (Reads Per Kilobase Transcriptome per Million mapped reads) values of the mapped genes. The differential expressed genes (DEGs) were identified using the absolute value of log2 (ratio) ≥1 as the threshold. Gene ontology (GO) annotation of genes was conducted with Blast2GO [56]. The enrichment analysis was carried out by GO:Termfinder using the hypergeometric test [57, 58] and P-values were corrected using the Bonferroni method [59].

### Validation of the RNA-seq analysis by qRT-PCR

A qRT-PCR process is typically used to confirm data obtained from high-throughput sequencing [60, 61]. In this study, 16 identified genes were used to verify the analysis results of transcriptional sequencing. Total RNA from the muscle tissue of the hybrid grouper and its parents were extracted and purified with the RNeasy Animal Mini Kit (Qiagen, Valencia, CA), and first-strand cDNA was obtained using a RevertAid First Strand cDNA Synthesis Kit (Fermentas, Vilnius, Lithuania). The qRT-PCR reactions based on SYBR (SYBR Green I, Osaka, Japan) were performed with a LightCycler 480 system (Roche, Basel, Switzerland) using the reaction procedure as follows: 95°C for 1 min followed by 40 cycles of 95°C for 10 s and 60°C for 30 s. All samples were examined in triplicate (i.e., three separate biological replicates) with β-actin as the internal control, and the 2^-ΔΔCt^ method [62] was used to calculate relative expression amounts. A standard curve was generated by running serial 10-fold dilutions (6 × 10^2^ to 6 × 10^8^) of a recombinant plasmid (pLB-β-actin) obtained as the template. Copy numbers of β-actin were detected to determine its stable and uniform expression in all samples. All gene-specific primers for target genes as well as the β-actin are summarized in the S1 Table.

## Results

### High-throughput sequencing and mapping to the reference genome

To obtain the muscle transcriptome data of the hybrid F1 and its parent non-hybrids (1.5 years was used as the approximate age for each fish to remove age difference) and to investigate the gene expression variations among the 3 grouper species, three individuals from each species were caught and a total of 9 RNA samples were isolated from muscle tissues. All RNA samples were of high-quality with a 28S/18S ratio ≥ 1.0 and RIN ≥ 7.0 after an RNA quality assessment. Three cDNA libraries were then constructed separately for the Hyb, Efu and Ela. Approximately 5 Gb of raw data for each sample were produced, and a total of 169 million paired-end clean reads were finally obtained after data filtration (Table 1). All transcriptome reads generated in this study have been submitted to the Sequence Read Archive (SRA) database of NCBI (Hyb: SRX1631685; Efu: SRX1626373; Ela: SRX1631646).

**Table 1:** Statistics of transcriptome data generated from the hybrid F1 and its parents.

Sample	Number of clean reads	Number of reads mapped to genome	Mapping rate^a^	Number of reads mapped to genes	Mapping rate^a^	Unique matches to genes	Mapping rate
**Hyb**	54,743,866	32,834,074	62.43%	29,300,448	54.06%	28,018,366	53.27%
**Ela**	55,310,310	33,702,316	60.93%	28,234,437	51.05%	26,878,024	48.59%
**Efu**	58,753,044	35,718,079	60.79%	31,762,641	55.71%	30,606,157	52.09%

^a ^Reads with ≤ 5 base mismatches were counted when mapped to the reference sequences.

The clean reads were subsequently aligned to the *E*. *coioides* genome (from our unpublished data) with SOAP aligner 2.0 [54]. We assembled the reference based on more than 100× coverage paired-end reads from libraries of 200-bp, 500-bp, 800-bp, 2-kb, 5-kb, 10-kb and 20-kb insert sizes to achieve a good genome assembly. Approximately 62.43% (Hyb), 60.93% (Ela) and 60.79% (Efu) of clean reads were mapped to the reference genome (≤ 5 base mismatches), respectively, in which 54.06% (Hyb), 51.05% (Ela) and 55.71% (Efu) of the reads were mapped to the gene regions (≤ 5 base mismatches). Moreover, 53.27% (Hyb), 48.59% (Ela) and 52.09% (Efu) of those uniquely mapped reads were further selected for gene quantification analysis (Table 1). Interestingly, all these mapping rates are higher than the previously published data on the liver and brain [21], which suggested that genes expressed in muscle may be more conserved than those in the other two tissues among the studied groupers.

### RPKM calculation and GO enrichment of DEGs

Gene expression levels were quantified by RPKM values. RPKMs of each gene in the muscle of the hybrid grouper were compared to its parents (S2 Table). The differentially expressed genes (DEGs) between the hybrid grouper and its parents were identified through filtering based on the criteria of false discovery rate (FDR) ≤0.05 and absolute log2 (ratio) ≥1. Our data showed that compared to Efu, there were 553 up-regulated and 1,250 down-regulated genes in the Hyb (S3 Table). Compared to Ela, the corresponding numbers of DEGs were 389 and 3,287, respectively (S4 Table). In total, there were 5,479 genes that were differentially expressed in the Hyb compared to its parents, and among those genes, 574 overlap DEGs were found in ‘Hyb vs. Efu’ and ‘Hyb vs. Ela’. Therefore, a total of 4,905 unique DEGs were identified and they were further selected for Gene Ontology (GO) enrichment analysis.

**Fig 1:**
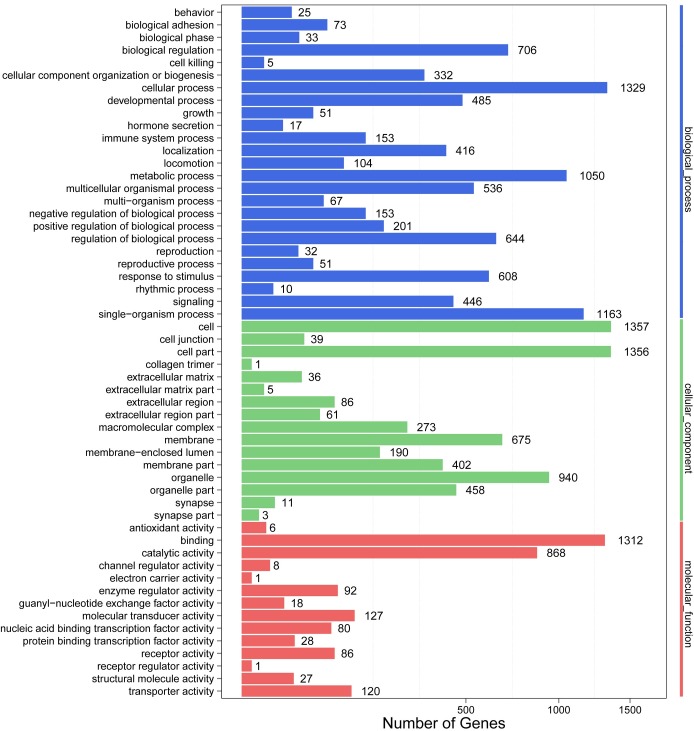
GO enrichment in the muscle transcriptome of the Hyb. The left y-axis indicates the identified GO terms. The right y-axis indicates the three groups of GO enrichment. The down x-axis represents the number of genes in certain category.

GO analysis of our dataset by Blast2GO [56] showed that all the above-mentioned 4,905 unique DEGs identified in the Hyb were enriched into 55 GO terms, in which cell and cell part were the top two enriched terms with 1,357 and 1,356 annotated genes, respectively (Fig 1). Among these 4,905 genes, 1,715 genes were grouped into biological process, 1,698 genes into cellular component and 1,771 genes into molecular function (Fig 1). In the liver, we previously found that metabolic process and catalytic activity were the most enriched processes out of the molecular function and biological process categories [21]. In the muscle, a large number of genes were also grouped into metabolic process (868 genes) and catalytic activity (1,050 genes), which made them the second and third most-enriched terms in each categories. The results suggested that extensive metabolic and catalytic activities in the muscles of the hybrid may also contribute to enhanced growth.

### Glycolytic genes are up-regulated in the hybrid grouper

We formerly showed that in the liver and brain the upstream GH/IGF axis and its downstream signaling pathways, such as glycogen synthesis, may be related to growth superiority in the hybrid grouper [21]. In this study, we further investigated whether in muscle, as one of the major tissues affected by the GH/IGF pathway, the genes in related downstream pathways (such as glycolysis) expressed differently between the hybrid and its parents.

**Fig 2:**
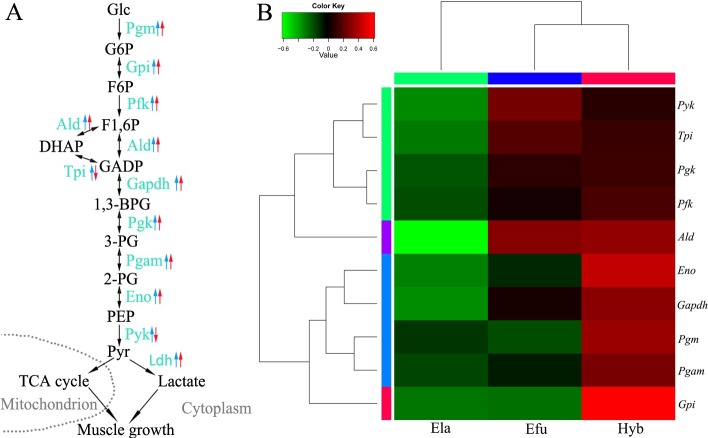
Most glycolysis genes were up-regulated in the hybrid grouper. (A) Diagram representing the 10 steps of glycolysis. Blue arrows denote the expression differences between the Hyb and paternal Ela. Red arrows denote the expression differences between the Hyb and maternal Efu. Up and down arrows stand for up- and down-regulation of transcription, respectively, in the Hyb; (B) Hierarchical cluster analysis of glycolytic genes. The color key represents RPKM values.

Glycolysis consists of 10 major steps with each reaction catalyzed by a specific enzyme. RPKM values of these 10 enzymes (Pgm, Gpi, Pfk, Ald, Tpi, Gapdh, Pgk, Pgam, Eno, Pyk) in the muscle of Hyb were compared to RPKM values of its parents Efu and Ela (Table 2). In the Hyb, we observed that all the 10 candidate genes had higher RPKMs than Ela with an excess number ranging from 280.91 (*Pfk*) to as high as 15,450.35 (*Ald*). Compared to Efu, up to 8 (*Pgm*, *Gpi*, *Pfk*, *Ald*, *Gapdh*, *Pgk*, *Pgam*, *Eno*) out of the 10 glycolytic genes were up-regulated in the Hyb, with an excess number ranging from 41.68 (*Pgk*) to as high as 7,002.71 (*Gapdh*). Consistent with this outcome, the RPKM of *Ldh*, which codes for the protein catalyzing the conversion of lactate to pyruvic acid, was much higher (6,245.80) in the Hyb than in the Efu (4,967.76) and Ela (4,986.57), which provides further evidence that the glycolysis pathway was activated in the Hyb (Fig 2). These genes may lead to active anabolism and catabolism in muscle of the Hyb and subsequently stimulate the development of the muscle in the hybrid grouper [34, 63].

**Table 2:** RPKMs of glycolytic genes in the three groupers.

GeneID	Gene name	Hyb-RPKM	Efu-RPKM	Ela-RPKM
ECO_PGM1_07035	*Pgm*	3,070.37	2,101.00	2,194.73
ECO_NA_20255	*Gpi*	3,228.49	1,770.03	1,755.65
ECO_NA_05450	*Pfk*	1,299.06	1,196.67	1,018.15
ECO_Q76BG1_ORYLA_15881	*Ald*	32,280.01	31,577.18	16,829.66
ECO_Q76BF5_ORYLA_00080	*Tpi*	4,868.45	5,110.67	3,636.00
ECO_GAPDH_00100	*Gapdh*	41,125.72	34,123.01	26,085.64
ECO_pgk1_01579	*Pgk*	1,535.34	1,493.66	1,204.62
ECO_PGAM2_13905	*Pgam*	10,060.13	7,849.86	7,349.46
ECO_ENO3_11835	*Eno*	14,821.15	10,080.36	8,788.70
ECO_NA_15670	*Pyk*	5,227.16	5,924.14	3,920.25

### Ca^2+^ signaling is activated and Tns were up-regulated in the Hyb

We further investigated, in addition to glycolysis, if genes in other pathways involved in muscle growth demonstrated different expression levels between the Hyb and its parents as well. Troponin (Tn) is a complex of three regulatory proteins (troponin C, troponin T and troponin I) that is integral to muscle contraction and development [46, 47]. Troponin C (TnC), containing four Ca^2+^-binding EF hands [42, 43], can directly bind to and sense Ca^2+^ [37, 44, 45], which was released into the cytosol by RyRs cellular calcium channels [38, 39]. We thus tested whether troponins and genes involved in the calcium signaling pathway were also up-regulated in the hybrid grouper.

**Fig 3:**
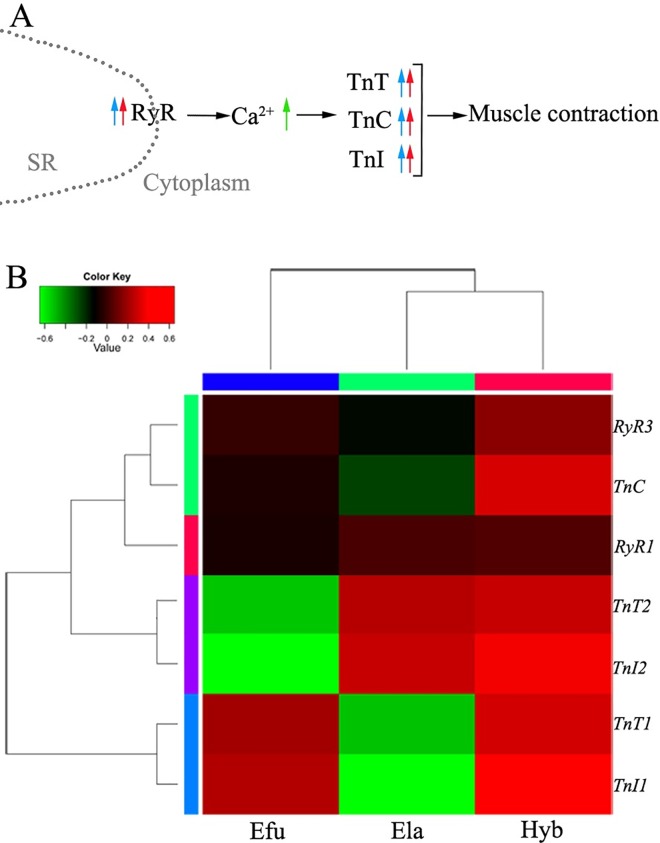
Ca^2+^ signal was activated by RyRs, with downstream troponin proteins up-regulated in the Hyb. (A) Diagram representing the related Ca^2+^ signaling pathway and the interaction between calcium and troponins. Blue arrows denote the expression differences between the Hyb and paternal Ela. Red arrows denote the expression differences between the Hyb and maternal Efu. Up arrows stand for up-regulation of transcription in the Hyb. The green up arrow denotes the increasing Ca^2+^ concentration in the cytoplasm. (B) Hierarchical cluster analysis of Ca^2+^ regulating genes and troponins. The color key represents RPKM values.

By comparing the RPKMs of Ca^2+^-regulating protein-coding genes in the Hyb and its parents (Table 3, Fig 3), RyRs (RyR1 and RyR3) were found to be up-regulated in the muscle of Hyb, which suggested there were active changes in the Ca^2+^ concentration of the cytosol in the cytoplasm. The downstream Ca^2+^-binding protein TnC was also up-regulated in the Hyb along with the other two troponins, TnI (TnI1, TnI2) and TnT (TnT1, TnT2) (Table 3, Fig 3). The activation of Ca^2+^ signaling as well as the abundance of the Tn complex may lead to severe muscle movement which facilitated the increase in the size of skeletal muscle [35].

**Table 3:** RPKMs of Ca2+ regulating genes and downstream troponins in the three groupers.

GeneID	Gene name	Hyb-RPKM	Efu-RPKM	Ela-RPKM
ECO_ryr1a_11089	*RyR1*	256.60	237.40	254.01
ECO_A4KUJ6_ORYLA_03678	*RyR3*	423.41	376.38	344.40
ECO_NA_06296	*TnC*	299.91	231.28	201.63
ECO_NA_04171	*TnI1*	398.77	357.04	193.70
ECO_NA_11258	*TnI2*	623.37	307.88	584.98
ECO_tnnt1_11276	*TnT1*	286.13	267.16	160.97
ECO_TNNT2_17228	*TnT2*	114.29	65.35	111.93

### Validation of the RNA-seq analysis by qRT-PCR

To further verify the expression levels of the above-mentioned genes in glycolysis and Ca^2+^ signaling pathways, qRT-PCR validation experiments were conducted using gene-specific primers (S1 Table), which were designed based on the mapped genome sequences. The efficiency of the primers was determined using PCR. A single band (ranging from approximately 150 bp to 300 bp in length) was observed in each lane, and the PCR products were sequenced and analyzed. The result showed that they were the target gene fragments. Standard curve analysis of β-actin showed R2 value of 0.99 and slope of -3.48, from which E (amplification efficiency) was calculated to be 94%, which is acceptable for qRT-PCR analysis. The calculation of copy numbers showed that β-actin exhibited stable and uniform expression in the three species, suggesting that it could be used as the housekeeping gene in this study.

**Fig 4:**
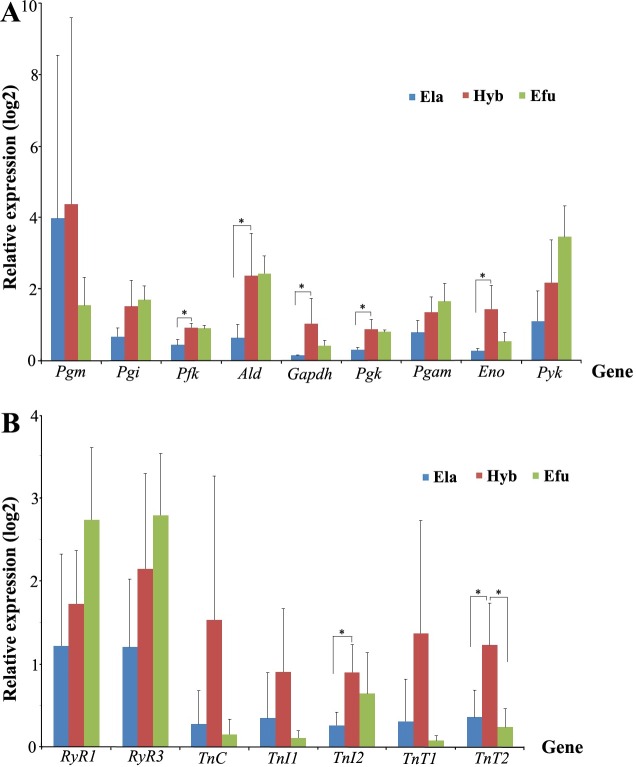
qRT-PCR validation of candidate genes. Nine glycolytic genes (A), RyR and Tn (B) were picked out as representatives of differences between the Hyb and its parents. β-actin was used as the internal control, and each value represents an average of three separate biological replicates. The error bars represent the standard deviation. Asterisk (*) was marked if there was significant difference between the two samples, as assessed by a t-test (*P* < 0.05).

The data showed that, consistent with the RNA-seq analysis, all glycolytic genes had higher expression in the Hyb than in Ela. Compared to Efu, *Pgm*, *Pfk*, *Gapdh*, *Pgk* and *Eno* were up-regulated in the hybrid grouper (Fig 4A). Regarding the troponins and genes involved in Ca^2+^ signaling pathway, all those genes had higher expressions in the Hyb compared to the Ela, whereas all troponins were up-regulated in the muscle of the Hyb compared to Efu (Fig 4B). These results confirmed that the expression differences of most studied transcripts among the 3 groupers revealed by both methods were generally consistent.

## Discussion

A novel grouper hybrid called the Hulong grouper (Hyb) exhibits better growth performance compared to its parents, Ela and Efu [10, 11]. In a previous study, we had inferred that the GH/IGF system and its downstream signaling pathways, such as glycogen synthesis, may contribute to growth superiority [21]. However, the underlying mechanism for muscle growth superiority in the Hyb is still unknown. Here, transcriptome analysis of muscle in Hulong grouper and its parents had been carried out, and we identified candidate genes regulating downstream glycolysis as well as muscle contraction involved Ca^2+^ signaling pathway and troponin synthesis. The qRT-PCR confirmed the differential expression in the selected genes. All of these differences, to a great degree, may account for the enhanced muscle growth of the hybrid.

### Glycolysis involved in muscle development

Glycolysis was reported to be involved in muscle development. Mutation of glycolytic genes, such as *Pfkm*, *Pgam* and *Pyk*, leads to different disease symptoms in muscle [34, 64]. Multiple muscle diseases have been known to show a reduction in glycolytic enzyme expression and an accumulation of glycogen [29–33]. In addition, reduced activity of glycolytic enzymes were observed in experimental animals with muscle-specific inactivation of proteins (such as mTOR) regulating cell growth [65], while significantly up-regulated expressions of glycolytic genes were detected in skeletal muscle-specific, conditional transgenic mice that expressed extra exogenic proteins (such as Akt1) stimulating muscle fiber growth [66]. In this study, almost all glycolytic genes were up-regulated in the muscle of the Hyb compared to its parents, which indicated that glycolysis may play an important role in the superior growth of the hybrid grouper.

Many metabolites in glycolysis are intermediates for other anabolic pathways as well, and as a consequence, the activation of the glycolysis pathway also led to the activation of multiple metabolic pathways, such as gluconeogenesis [67, 68], lipid metabolism [69], the citric acid cycle (CAC, also known as tricarboxylic acid or TCA cycle, a post-glycolytic process leading to amino acid synthesis, nucleotide synthesis and tetrapyrrole synthesis) [70, 71], among other processes. Therefore, the up-regulated expression of glycolytic enzymes may synchronously influence growth superiority in the Hyb not just via glycolysis, but in a much wider scope.

**Fig 5:**
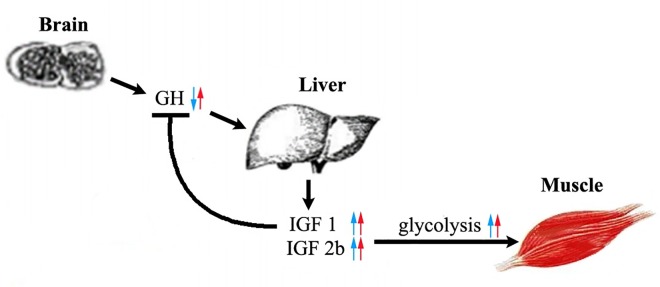
The GH/IGF axis may serve as the upstream pathway of glycolysis to stimulate enhanced growth. Blue arrows denote the expression differences between the Hyb and paternal Ela. Red arrows denote the expression differences between the Hyb and maternal Efu. Up and down arrows represent for up- and down-regulation of transcription, respectively, in the Hyb.

Glycolysis is a downstream pathway of the GH/IGF axis [72, 73]. Our previous study suggested that variation of GH/IGF system in the brain and liver may be the important cause of enhanced growth in the Hyb [21]. In our current study, we confirmed that in muscle, a target of the GH/IGF system, the downstream glycolysis was also activated in the Hyb with up-regulation of almost all glycolytic genes, which provided evidence for a possible critical role in the upstream GH/IGF pathway in muscle development by stimulating downstream glycolysis in the muscle as the major effector organ (Fig 5).

### Ca^2+^ signaling pathway and calcium-binding protein TnC

Calcium acts as a signal messenger combining excitation events with downstream effects [74]. Muscle contraction results from the binding of Ca^2+^ to a calcium-binding protein TnC in skeletal muscle [75, 76]. TnC, in addition to TnI and TnT, forms the troponin complex that binds to actin and tropomyosin [75]. The release of Ca^2+^ into the cytosol from SR via the RyR receptor channels raised the Ca^2+^ concentration and its binding to TnC changes the conformation of TnC, which ultimately resulted in the strong interaction between actin and myosin to generate force and muscle shortening [77]. In the muscle of the Hyb, Ca^2+^ signaling was activated and troponins were up-regulated, which suggested there was an active state of muscle contraction that led to fiber growth [35]. These data were consistent with the active swimming of the Hyb.

## An overall view on the the superior growth of the Hyb

**Fig 6:**
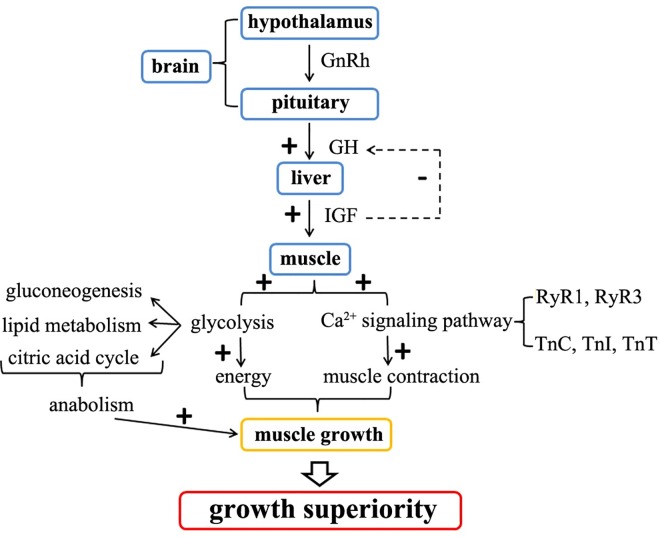
An overall view of the molecular mechanisms underlying enhanced growth of the Hyb. “+” and “-” stands for positive and negative regulation, respectively.

The heterosis of the hybrid Hyb grouper is represented in many aspects, such as faster growth rate and stronger disease resistance [10, 11]. Although the biological and physiological features of the Hyb have been well-studied, the genetic mechanisms of its heterosis, especially enhanced growth, had yet to be investigated. We formerly obtained the transcriptome sequences of the tissues in the neuroendocrine system (brain and liver) from the Hyb and its parents. Differential gene expression was observed in the GH/IGF axis and downstream signaling pathways, such as protein synthesis and glycogen synthesis [21]. In this study, we sequenced the transcriptomes of the major effector organ (muscle) from these three groupers, and the downstream glycolysis pathway was shown to be highly activated in the Hyb. In addition, the Ca^2+^ signaling pathway was activated via up-regulated RyRs (RyR1 and RyR3) and troponins (TnC, TnI and TnT), which resulted in elevation of muscle contraction and subsequently promoted muscle growth and body development (Fig 6).

## Conclusions

The novel hybrid grouper, called the Hulong grouper (Hyb), exhibits better growth performance and stronger disease resistance than its parents Efu (♀) and Ela (♂). Using the RNA-seq technique, we obtained the muscle transcripts from these 3 species. The transcriptomic data showed that almost all glycolytic enzymes were up-regulated in the Hyb. Troponins as well as genes involved in Ca^2+^ signaling were also up-regulated. The RNA expression levels of candidate genes were further validated by qRT-PCR. These findings revealed that glycolysis in addition to calcium signaling and up-regulated troponins may be the most important contributors to the growth superiority of the hybrid grouper.

## Supporting Information

S1 Table Sequences of specific primers for the qRT-PCR. (XLSX)

S2 Table RPKM values of all genes identified in the hybrid grouper and its parents. (XLSX)

S3 Table Differentially expressed genes in the muscle between Hyb and Efu. (XLSX)

S4 Table Differentially expressed genes in the muscle between Hyb and Ela. (XLSX)

## Abbreviations

Ald: fructose-bisphosphate aldolase
Eno: enolase
Gapdh: glyceraldehyde phosphate dehydrogenase
GH: growth hormone
Gpi: phosphoglucose isomerase
IGF: insulin-like growth factor
Ldh: lactate dehydrogenase
Pfk: phosphofructokinase
Pgam: phosphoglycerate mutase
Pgk: phosphoglycerate kinase
Pgm: phosphoglucomutase
Pyk: pyruvate kinase
RyR: ryanodine receptor
SR: sarcoplasmic reticulum
Tn: troponin
TnC: troponin C
TnI: troponin I
TnT: troponin T
Tpi: triosephosphate isomerase

## References

[1] Guo CY, Huang YH, Wei SN, Ouyang ZL, Yan Y, Huang XH, et al Establishment of a new cell line from the heart of giant grouper, *Epinephelus lanceolatus* (Bloch), and its application in toxicology and virus susceptibility. J Fish Dis. 2015; 38(2): 175–186. 10.1111/jfd.12221 24372271

[2] Pierre S, Gaillard S, Prévot-D'Alvise N, Aubert J, Rostaing-Capaillon O, Leung-Tack D, et al Grouper aquaculture: asian success and mediterranean trials. Aquatic Conserv: Mar Freshw Ecosyst. 2008; 18(3): 297–308.

[3] Kiriyakit A, Gallardo WG, Bart AN. Successful hybridization of groupers (*Epinephelus coioides* x *Epinephelus lanceolatus*) using cryopreserved sperm. Aquaculture. 2011; 320(1): 106–112.

[4] Tseng WY, Poon CT. Hybridization of Epinephelus species. Aquaculture. 1983; 34(1): 177–182.

[5] James CM, Al-Thobaiti SA, Rasem BM, Carlos MH. Potential of grouper hybrid (*Epinephelus fuscoguttatus* x *E*. *polyphekadion*) for aquaculture. Naga, the ICLARM Quarterly. 1999; 22(1): 19–23.

[6] Glamuzina B, Kozul V, Tutman P, Skaramuca B. Hybridization of mediterranean groupers: *Epinephelus marginatus*♀ × *E*. *aeneus*♂ and early development. Aquacult Res. 1999; 30(8): 625–628.

[7] Glamuzina B, Glavić N, Skaramuca B, Kozul V, Tutman P. Early development of the hybrid *Epinephelus costae*♀ × *E*. *marginatus*♂. Aquaculture. 2001; 198(1): 55–61.

[8] Liufu YZ, Zhao HZ, Liu XC, Lin HR, Huang GG, Zhang HF, et al Preliminary study on he hybrid red-spotted grouper (*Epinephelus akaara*)♂ × orange-spotted grouper (*Epinephelus coioides*)♀. Acta Scientiarum Maturalium Universitatis Sunyatseni. 2007; 46(3): 72–75.

[9] Wang Y, Zhang Y, Zhang HF, Huang W, Xiao L, Li SS, et al Comparison of morphology between two kinds of hybrid groupers and their parents. Journal of Fisheries of China. 2014; 38: 778–785.

[10] Zhou H. Genetic analysis for two grouper hybrids and their parents. Master degree thesis, Sun Yat-sen University, Guangzhou. 2011; 1–2.

[11] Senoo S. Hybrid production between tiger grouper *Epinephelus Fuscoguttatus* x giant grouper *Epinephelus Lanceolatus*. Aquanet Magazine. 2006; 12: 58–63.

[12] Chen C, Kong XD, Li YL, Song ZX, Jia RJ, Yu HH, et al Embryonic and morphological development in the larva, juvenile and young stages of *Epinephelus fuscoguttatus*♀ and *E*. *lanceolatus*♂. Progress in Fishery Sciences. 2014; 35 (5): 135–144.

[13] Yu HH, Li YL, Chen C, Kong XD, Zhang TT, Liu L, et al Feeding habits and growth characteristics of larval, juvenile, and young F1 of *Epinephelus fuscoguttatus* (♀) x *E*. *lanceolatus* (♂). Journal of Fishery Sciences. 2015; 22(5): 968–977.

[14] Fuentes EN, Valdes JA, Molina A, Bjornsson BT. Regulation of skeletal muscle growth in fish by the growth hormone-insulin-like growth factor system. Gen Comp Endocrinol. 2013; 192: 136–148. 10.1016/j.ygcen.2013.06.009 23791761

[15] Rowe RW, Goldspink G. Muscle fibre growth in five different muscles in both sexes of mice. J Anat. 1969; 104(3): 519–530. 5804561 PMC1231952

[16] Rehfeldt C, Fiedler I, Dietl G, Ender K. Myogenesis and postnatal skeletal muscle cell growth as influenced by selection. Livest Prod Sci. 2000; 66(2): 177–188.

[17] Timson BF, Dudenhoeffer GA. Skeletal muscle fibre number in the rat from youth to adulthood. J Anat. 1990; 173: 33–36. 2074227 PMC1256077

[18] Stickland NC. Growth and development of muscle fibres in the rainbow trout (*Salmo gairdneri*). J Anat. 1983; 137: 323–333. 6630043 PMC1171824

[19] Weatherley A, Gill H, Lobo A. Recruitment and maximal diameter of axial muscle fibres in teleosts and their relationship to somatic growth and ultimate size. J Fish Biol. 1988; 33(6): 851–859.

[20] Weatherley A, Gill H. Dynamics of increase in muscle fibers in fishes in relation to size and growth. Experientia. 1985; 41(3): 353–354.

[21] Sun Y, Guo CY, Wang DD, Li XF, Xiao L, Zhang XH, et al Transcriptome analysis reveals the molecular mechanisms underlying growth superiority in a novel grouper hybrid (*Epinephelus fuscogutatus*♀ x *E*. *lanceolatus*♂). BMC Genet. 2016; 17(1): 1. 26785614 10.1186/s12863-016-0328-y PMC4719697

[22] Sotiropoulos A, Ohanna M, Kedzia C, Menon RK, Kopchick JJ, Kelly PA, et al Growth hormone promotes skeletal muscle cell fusion independent of insulin-like growth factor 1 up-regulation. PNAS. 2006; 103(19): 7315–7320. 10.1073/pnas.0510033103 16670201 PMC1456062

[23] Short KR, Moller N, Bigelow ML, Coenen-Schimke J, Nair KS. Enhancement of muscle mitochondrial function by growth hormone. Journal of Clinical Endocrinology and Metabolism. 2008; 93(2): 597–604. 10.1210/jc.2007-1814 18000087 PMC2243230

[24] Fothergill-Gilmore LA, Michels PA. Evolution of glycolysis. Prog Biophys Mol Biol. 1993; 59(2): 105–235. 8426905 10.1016/0079-6107(93)90001-z

[25] Baanante IV, Garcia de Frutos P, Bonamusa L, Fernandez F. Regulation of fish glycolysis-gluconeogenesis: role of fructose 2,6 P2 and PFK-2. Comp Biochem Physiol B. 1991; 100(1): 11–17.

[26] Carracedo A, Cantley LC, Pandolfi PP. Cancer metabolism: fatty acid oxidation in the limelight. Nat Rev Cancer. 2013; 13(4): 227–232. 10.1038/nrc3483 23446547 PMC3766957

[27] Sharma N, Okere IC, Brunengraber DZ, McElfresh TA, King KL, Sterk JP, et al Regulation of pyruvate dehydrogenase activity and citric acid cycle intermediates during high cardiac power generation. J Physiol. 2005; 562(2): 593–603. 15550462 10.1113/jphysiol.2004.075713 PMC1665507

[28] Greiner EF, Guppy M, Brand K. Glucose is essential for proliferation and the glycolytic enzyme induction that provokes a transition to glycolytic energy production. J Biol Chem. 1994; 269(50): 31484–31490. 7989314

[29] Parker KC, Kong SW, Walsh RJ, Salajegheh M, Moghadaszadeh B, Amato AA, et al Fast-twitch sarcomeric and glycolytic enzyme protein loss in inclusion body myositis. Muscle Nerve. 2009; 39(6): 739–753. 10.1002/mus.21230 19291799 PMC2753483

[30] Coley W, Rayavarapu S, Pandey GS, Sabina RL, Van der Meulen JH, Ampong B, et al The molecular basis of skeletal muscle weakness in a mouse model of inflammatory myopathy. Arthritis Rheum. 2012; 64(11): 3750–3759. 10.1002/art.34625 22806328 PMC3485437

[31] Sharma U, Atri S, Sharma MC, Sarkar C, Jagannathan NR. Skeletal muscle metabolism in Duchenne muscular dystrophy (DMD): an in-vitro proton NMR spectroscopy study. Magn Reson Imaging. 2003; 21(2): 145–153. 12670601 10.1016/s0730-725x(02)00646-x

[32] Lodi R, Kemp GJ, Muntoni F, Thompson CH, Rae C, Taylor J, et al Reduced cytosolic acidification during exercise suggests defective glycolytic activity in skeletal muscle of patients with Becker muscular dystrophy. An in vivo 31P magnetic resonance spectroscopy study. Brain. 1999; 122(1): 121–130. 10050900 10.1093/brain/122.1.121

[33] Servidei S, Bonilla E, Diedrich RG, Kornfeld M, Oates JD, Davidson M, et al Fatal infantile form of muscle phosphofructokinase deficiency. Neurology. 1986; 36(11): 1465–1470. 2945125 10.1212/wnl.36.11.1465

[34] Tixier V, Bataille L, Etard C, Jagla T, Weger M, Daponte JP, et al Glycolysis supports embryonic muscle growth by promoting myoblast fusion. Proc Natl Acad Sci USA. 2013; 110(47): 18982–18987. 10.1073/pnas.1301262110 24191061 PMC3839714

[35] Baar K, Esser K. Phosphorylation of p70S6kcorrelates with increased skeletal muscle mass following resistance exercise. Am J Phys-Cell Ph. 1999; 276(1): C120–C127. 10.1152/ajpcell.1999.276.1.C120 9886927

[36] Tidball JG. Mechanical signal transduction in skeletal muscle growth and adaptation. J Appl Physiol. 2005; 98(5): 1900–1908. 10.1152/japplphysiol.01178.2004 15829723

[37] Herzberg O, Moult J, James MN. Calcium binding to skeletal muscle troponin C and the regulation of muscle contraction. Ciba Found Symp. 1985; 122: 120–144. 10.1002/9780470513347.ch8 3792134

[38] Coronado R, Morrissette J, Sukhareva M, Vaughan DM. Structure and function of ryanodine receptors. Am J Physiol. 1994; 266(6): 1485–1504. 10.1152/ajpcell.1994.266.6.C1485 8023884

[39] Protasi F, Franzini-Armstrong C, Allen PD. Role of ryanodine receptors in the assembly of calcium release units in skeletal muscle. J Cell Biol. 1998; 140(4): 831–842. 9472035 10.1083/jcb.140.4.831 PMC2141739

[40] Yamaguchi F, Umeda Y, Shimamoto S, Tsuchiya M, Tokumitsu H, Tokuda M, et al S100 proteins modulate protein phosphatase 5 function: a link between CA2+ signal transduction and protein dephosphorylation. J Biol Chem. 2012; 287(17): 13787–13798. 10.1074/jbc.M111.329771 22399290 PMC3340197

[41] Clapham DE. Calcium signaling. Cell. 2007; 131(6): 1047–1058. 10.1016/j.cell.2007.11.028 18083096

[42] Li AY, Lee J, Borek D, Otwinowski Z, Tibbits GF, Paetzel M. Crystal structure of cardiac troponin C regulatory domain in complex with cadmium and deoxycholic acid reveals novel conformation. J Mol Biol. 2011; 413(3): 699–711. 10.1016/j.jmb.2011.08.049 21920370 PMC4068330

[43] Grabarek Z. Insights into modulation of calcium signaling by magnesium in calmodulin, troponin C and related EF-hand proteins. Biochim Biophys Acta. 2011; 1813(5): 913–921. 10.1016/j.bbamcr.2011.01.017 21262274 PMC3078997

[44] Polyakov AA, Gusev NB. Utilization of troponin C as a model calcium-binding protein for mapping of the calmodulin-binding sites of caldesmon. Biochem J. 1997; 321(3): 873–878. 9032478 10.1042/bj3210873 PMC1218147

[45] Herzberg O, James MN. Structure of the calcium regulatory muscle protein troponin-C at 2.8 A resolution. Nature. 1985; 313(6004): 653–659. 3974698 10.1038/313653a0

[46] Lehman W, Galinska-Rakoczy A, Hatch V, Tobacman LS, Craig R. Structural basis for the activation of muscle contraction by troponin and tropomyosin. J Mol Biol. 2009 388(4): 673–681. 10.1016/j.jmb.2009.03.060 19341744 PMC2693027

[47] Gomes AV, Potter JD, Szczesna-Cordary D. The role of troponins in muscle contraction. IUBMB Life. 2002; 54(6): 323–333. 10.1080/15216540216037 12665242

[48] Brennan C, Mangoli M, Dyer CE, Ashworth R. Acetylcholine and calcium signalling regulates muscle fibre formation in the zebrafish embryo. J Cell Sci. 2005; 118(22): 5181–5190. 16249237 10.1242/jcs.02625

[49] Jurynec MJ, Xia R, Mackrill JJ, Gunther D, Crawford T, Flanigan KM. Selenoprotein N is required for ryanodine receptor calcium release channel activity in human and zebrafish muscle. Proc Natl Acad Sci USA. 2008; 105(34): 12485–12490. 10.1073/pnas.0806015105 18713863 PMC2527938

[50] Sehnert AJ, Huq A, Weinstein BM, Walker C, Fishman M, Stainier DY. Cardiac troponin T is essential in sarcomere assembly and cardiac contractility. Nat Genet. 2002; 31(1): 106–110. 10.1038/ng875 11967535

[51] Ferrante MI, Kiff RM, Goulding DA, Stemple DL. Troponin T is essential for sarcomere assembly in zebrafish skeletal muscle. J Cell Sci. 2011; 124(4): 565–577. 21245197 10.1242/jcs.071274 PMC3031369

[52] Sogah VM, Serluca FC, Fishman MC, Yelon DL, MacRae CA, Mably JD. Distinct troponin c isoform requirements in cardiac and skeletal muscle. Dev Dyn. 2010; 239(11): 3115–3123. 10.1002/dvdy.22445 20925115 PMC2965274

[53] Short Oligonucleotide Analysis Package, http://soap.genomics.org.cn/.

[54] Luo R, Liu B, Xie Y, Li Z, Huang W, Yua J, et al SOAPdenovo2: an empirically improved memory-efficient short-read de novo assembler. Gigascience. 2012; 1(1): 18 10.1186/2047-217X-1-18 23587118 PMC3626529

[55] Trapnell C, Williams BA, Pertea G, Mortazavi A, Kwan G, Baren MJ, et al Transcript assembly and abundance estimation from RNA-Seq reveals thousands of new transcripts and switching among isoforms. Nat Biotechnol. 2010; 28(5): 511–515. 20436464 10.1038/nbt.1621 PMC3146043

[56] Conesa A, Götz S, García-Gómez JM, Terol J, Talón M, Robles M. Blast2GO: a universal tool for annotation, visualization and analysis in functional genomics research. Bioinformatics. 2005; 21(18): 3674–3676. 10.1093/bioinformatics/bti610 16081474

[57] Flight RM, Wentzell PD. Potential bias in GO::TermFinder. Brief Bioinform. 2009; 10(3): 289–294. 10.1093/bib/bbn054 19279157

[58] Boyle EI, Weng S, Gollub J, Jin H, Botstein D, Cherry JM, et al GO::TermFinder—open source software for accessing Gene Ontology information and finding significantly enriched Gene Ontology terms associated with a list of genes. Bioinformatics. 2004; 20(18): 3710–3715. 10.1093/bioinformatics/bth456 15297299 PMC3037731

[59] Abdi H. The Bonferonni and Šidák corrections for multiple comparisons. Encycl Meas Stat. 2007; 3: 103–107.

[60] Chen S, Yang P, Jiang F, Wei Y, Ma Z, Kang L. De novo analysis of transcriptome dynamics in the migratory locust during the development of phase traits. PloS One. 2010; 5(12): e15633 10.1371/journal.pone.0015633 21209894 PMC3012706

[61] Kalavacharla V, Liu Z, Meyers BC, Thimmapuram J, Melmaiee K. Identification and analysis of common bean (*Phaseolus vulgaris L*.) transcriptomes by massively parallel pyrosequencing. BMC Plant Biol. 2011; 11(1): 1. 21985325 10.1186/1471-2229-11-135 PMC3209450

[62] Livak KJ, Schmittgen TD. Analysis of relative gene expression data using real-time quantitative PCR and the 2^-ΔΔCt^ Method. Methods. 2001; 25(4): 402–408. 10.1006/meth.2001.1262 11846609

[63] Godfrey R, Quinlivan R. Skeletal muscle disorders of glycogenolysis and glycolysis. Nat Rev Neurol. 2016; 12(7): 393–402. 10.1038/nrneurol.2016.75 27231184

[64] Garcia M, Pujol A, Ruzo A, Riu E, Ruberte J, Arbos A, et al Phosphofructo-1-kinase deficiency leads to a severe cardiac and hematological disorder in addition to skeletal muscle glycogenosis. PLoS Genet. 2009; 5(8): e1000615 10.1371/journal.pgen.1000615 19696889 PMC2721631

[65] Risson V, Mazelin L, Roceri M, Sanchez H, Moncollin V, Corneloup C, et al Muscle inactivation of mTOR causes metabolic and dystrophin defects leading to severe myopathy. J Cell Biol. 2009; 187(6): 859–874. 10.1083/jcb.200903131 20008564 PMC2806319

[66] Izumiya Y, Hopkins T, Morris C, Sato K, Zeng L, Viereck J, et al Fast/Glycolytic muscle fiber growth reduces fat mass and improves metabolic parameters in obese mice. Cell Metab. 2008; 7(2): 159–172. 10.1016/j.cmet.2007.11.003 18249175 PMC2828690

[67] Diamant YZ, Shafrir E. Placental enzymes of glycolysis, gluconeogenesis and lipogenesis in the diabetic rat and in starvation. Comparison with maternal and foetal liver. Diabetologia. 1978; 15(6): 481–485. 720782 10.1007/BF02342874

[68] Cohen GN. Glycolysis, gluconeogenesis and glycogen and cellulose synthesis Microbial Biochemistry. Springer Netherlands 2016; pp.107–122.

[69] Hopperton KE, Duncan RE, Bazinet RP, Archer MC. Fatty acid synthase plays a role in cancer metabolism beyond providing fatty acids for phospholipid synthesis or sustaining elevations in glycolytic activity. Exp Cell Res. 2014; 320(2): 302–310. 10.1016/j.yexcr.2013.10.016 24200503

[70] Panchal AR, Comte B, Huang H, Dudar B, Roth B, Chandler M, et al Acute hibernation decreases myocardial pyruvate carboxylation and citrate release. Am J Physiol Heart Circ Physiol. 2001; 281(4): 1613–1620. 10.1152/ajpheart.2001.281.4.H1613 11557551

[71] Lee SH, Davis EJ. Carboxylation and decarboxylation reactions. Anaplerotic flux and removal of citrate cycle intermediates in skeletal muscle. J Biol Chem. 1979; 254(2): 420–430. 762069

[72] Noguchi R, Kubota H, Yugi K, Toyoshima Y, Komori Y, Soga T, et al The selective control of glycolysis, gluconeogenesis and glycogenesis by temporal insulin patterns. Mol Syst Biol. 2013; 9(1): 664. 23670537 10.1038/msb.2013.19 PMC4039368

[73] Probst I, Unthan-Fechner K. Activation of glycolysis by insulin with a sequential increase of the 6-phosphofructo-2-kinase activity, fructose-2,6-bisphosphate level and pyruvate kinase activity in cultured rat hepatocytes. Eur J Biochem. 1985; 153(2): 347–353. 3000776 10.1111/j.1432-1033.1985.tb09309.x

[74] Berchtold MW, Brinkmeier H, Muntener M. Calcium ion in skeletal muscle: its crucial role for muscle function, plasticity, and disease. Physiol Rev. 2000; 80(3): 1215–1265. 10893434 10.1152/physrev.2000.80.3.1215

[75] Farah CS, Reinach FC. The troponin complex and regulation of muscle contraction. Faseb J. 1995; 9(9): 755–767. 7601340 10.1096/fasebj.9.9.7601340

[76] Filatov VL, Katrukha AG, Bulargina TV, Gusev NB. Troponin: structure, properties, and mechanism of functioning. Biochemistry. 1999; 64: 969–985. 10521712

[77] Gordon AM, Homsher E, Regnier M. Regulation of contraction in striated muscle. Physiol Rev. 2000; 80(2): 853–924. 10747208 10.1152/physrev.2000.80.2.853

